# Digestibility of Coffee and Sugar

**Published:** 1883-03

**Authors:** 


### DIGESTIBILITY OF COFFEE AND SUGAR.

M. Leved, in *La Mėdecin Practicien*, gives the results of some experimental investigations made to determine the degree of digestibility of coffee and sugar. To a dog which had eaten 210 grammes of meat he administered 30 grains of coffee in 15 grammes of water. After three hours he killed the dog, and found the mucous membrane of the stomach pale, discolored and profoundly anæmic. The vessels of the internal superficies, like those of the periphery were contracted; 145 grammes of the meat remained undigested; the coffee then had retarded the stomach digestion. The abuse of coffee produces dyspepsia; the English and Hollanders, who take coffee and tea in large quantities, are frequently dyspeptic. Coffee elevates the cerebral functions; it has a pleasant and useful general effect, but the local effect is *generally* bad. M. Leved does not agree with physicians and medical chemists, who think that sugar is hurtful to dyspeptics; citing the case of one of his friends, a dyspeptic for two years, who did not take sugar, and was afraid of it, but he commenced taking 120 grammes daily without the slightest bad effect. Having given a dog 80 grammes of sugar after he had eaten 200 grammes of meat, he killed him after six hours, and found no meat in the stomach. The mucous membrane was red and widely congested. The hyperæmia of the liver was marked. After killing the dog which had eaten 200 grammes of meat and *no* sugar, he found 90 or 100 grammes still undigested. Sugar favors the secretion of gastric juice. Sugared coffee is unfavorable to it.

